# Psychological impact of telework during the COVID-19 pandemic in Tunisia

**DOI:** 10.1192/j.eurpsy.2023.1725

**Published:** 2023-07-19

**Authors:** W. Ayed, I. Aissa, S. Chebbi, A. Ayadi, I. Magroun

**Affiliations:** 1 Occupational health departement, University of Tunis El Manar - faculty of Medicine of Tunis, Ariana; 2 Occupational health departement, University of Tunis El Manar - faculty of Medicine of Tunis, nabeul, Tunisia

## Abstract

**Introduction:**

Telework is a work organization in which a task that could have been performed on the employer’s worksite is performed by an employee outside of these offices, using information and communication technologies. COVID-19 pandemic has strengthened digitalization as the result of social distancing and lockdown. However, teleworking can lead to different risks for employees mental health.

**Objectives:**

Evaluation of the psychosocial impact of telework during the first wave of the COVID-19 pandemic in Tunisia

**Methods:**

Descriptive cross-sectional study carried out from17 to 22 May 2021. It included workers who teleworked during the first lockdown. The data collection was performed with a self-administered online questionnaire specifying the socio-medical, occupational and psychosocial characteristics.

**Results:**

A total of 612 teleworkers were included. The mean age was 33±6.9 years. Sex ratio (M/F) was 0.32. The main sectors of activity were telecommunications and information technology (31.6%), legal and financial services (19%) and administration and organizations (16.5%). The teleworkers were operating in the private sector in 91.6% of the cases. Teleworking had been practiced before the health confinement by 55.6% of the cases and 86.3% had never received teleworking training. Psychosocial repercussions were noticed among 92.2%. During confinement, teleworkers reported a mood sadness in 36.4%, persistent anxiety in 27.8% and constant exhaustion in 43.3%. Sleep disorders were reported by 65.5%. They were difficulty in getting to sleep in 42.5% and a difficult morning awakening in 51.8%. The absence of work organization was significantly correlated with mood sadness (p<0.001), chronic anxiety (p=0.01), sleep disorders (p=0.03), and constant exhaustion (p=0.001). Spending breaks in front of the television and on social networks was significantly correlated with sadness of mood (p=0.04), anxiety (p=0.009), and sleep disorders (p=0.04).

**Conclusions:**

Psychological impact of teleworking during health confinement at the COVID-19 pandemic was significant. Therefore, the role of the occupational physician is important in the detection and the prevention of health consequences.

**Disclosure of Interest:**

None Declared

